# *Streptococcus pneumoniae* Serotype 19A in Children, South Korea

**DOI:** 10.3201/eid1402.070807

**Published:** 2008-02

**Authors:** Eun Hwa Choi, So Hee Kim, Byung Wook Eun, Sun Jung Kim, Nam Hee Kim, Jina Lee, Hoan Jong Lee

**Affiliations:** * Seoul National University College of Medicine, Seoul, South Korea; † Seoul National University Bundang Hospital, Seongnam, South Korea; ‡ Inje University College of Medicine, Goyang, South Korea; § Seoul National University Boramae Hospital, Seoul, South Korea; ¶ Seoul National University Hospital, Seoul, South Korea

**Keywords:** *Streptococcus pneumoniae*, serotype 19A, multilocus sequence typing, pneumococcal conjugate vaccine, research

## Abstract

Despite the concern of replacement disease, notably by serotype 19A after 7-valent conjugate vaccine (PCV7) use, serotype 19A was increasingly recognized in Korean children before the introduction of PCV7. To understand the dynamics of serogroup 19 prevalence from 1991–2006, we serotyped 538 pediatric pneumococcal isolates. Serogroup 19 isolates (n = 126) were characterized by antimicrobial drug susceptibility, presence of *mef*A/*erm*B, and multilocus sequence typing. Overall, the proportion of serotype 19A isolates increased but serotype 19F decreased. Among children <5 years of age, the proportion of serotype 19A isolates in invasive pneumococcal disease increased from 0% in 1991–1994 to 8%–10% in 1995–2000, reached 26% in 2001–2003, and remained at 20% in 2004–2006 when vaccine coverage did not exceed 25% (p = 0.005 for trend). This study demonstrates that the expansion of multidrug-resistant ST320 was responsible for the increase in serotype 19A before PCV7 use.

## Abstract 2

A single, multidrug-resistant strain was responsible for increased incidence of this serotype before introduction of the pneumococcal 7-valent conjugate vaccine.

*Streptococcus pneumoniae* is a major cause of invasive infections in young infants and children. Among >90 serotypes, only a limited number account for pneumococcal diseases. Serotype incidence can vary by patient age, geographic region, and time of surveillance. Since the introduction of 7-valent conjugate vaccine (PCV7) in the United States, a decrease in the incidence of invasive pneumococcal disease (IPD) caused by vaccine serotypes has been observed in pediatric and nonpediatric age groups (*1**,**2*). However, the incidence of IPD caused by nonvaccine serotypes (including serotype 19A) increased 1.5-fold in 2002 compared to that in 1999 (*2**,**3*). To date, replacement for IPD has been observed for serotypes 3, 15, 19A, 22F, 33F, and 35, with the increase in 19A being the most prevalent. (*2**–**7*). Recently, Singleton et al. reported that serotype 19A was responsible for 28.3% of IPD in rural Alaska Native children <2 years of age during 2004–2006 (*8*). In addition, recent studies from Massachusetts and Texas showed that a multidrug-resistant sequence type of serotype 19A has emerged as an important cause of IPD (*9**,**10*).

The direct effects of PCV7 in serotype distribution changes and genetic structures of pneumococcal isolates are not clear. However, capsular switching of vaccine serotypes under selective pressure by PCV7 is one of the mechanisms underlying the expansion of serotype 19A (*4**,**11**–**13*).

As part of this surveillance implemented in a tertiary referral center in South Korea, all pneumococcal isolates obtained from sterile body fluids and from various clinical specimens were subjected to serotype and antimicrobial drug susceptibility pattern determinations (*14*). Surveillance since 1991 shows that serotype 19A isolates were increasingly recognized among clinical isolates before PCV7 was introduced in South Korea in November 2003. We describe *S. pneumoniae* serotype distribution changes in Korean children during 1991–2006 with an emphasis on serogroup 19.

## Methods

### Patients and Pneumococcal Isolates

Hospital-wide surveillance was continued to monitor pneumococcal diseases as a part of routine clinical care at Seoul National University Children’s Hospital from 1991 through 2006. Only initial isolates were included in the study; repeated isolates from the same patient were excluded. Isolates were classified as invasive (e.g., blood, pleural fluid, cerebrospinal fluid, joint fluid) and noninvasive (pharynx, middle ear fluid, sputum). Methods of surveillance and of obtaining blood cultures did not change during the study period.

### Analysis of Serotype Distributions and Classifications

All isolates were serotyped by the Quellung reaction using antiserum (Statens Serum Institute, Copenhagen, Denmark). The study period was divided into five 3- or 4- year-periods: 1991–1994 (period 1), 1995–1997 (period 2), 1998–2000 (period 3), 2001–2003 (period 4), and 2004–2006 (period 5). The PCV7 serotypes were 4, 6B, 9V, 14, 18C, 19F, and 23F. Serotypes considered to be PCV7 related included those not directly targeted by PCV7 but of the same serogroups (6A, 9A, 9N, 18B, 18F, and 23A); serotype 19A was analyzed separately. Non-PCV7 serotypes included all other serotypes.

### Multilocus Squence Typing Analysis

Multilocus sequence typing (MLST) was performed on all serotype 19A isolates (n = 58) and serotype 19F isolates (n = 68) obtained from children <5 years of age (*15**–**17*). New alleles were verified by resequencing gene fragments of both strands. When sequence types (STs) that had not been associated with a particular serotype in the past were identified, serotyping and sequencing typing were confirmed by repeating the reactions. STs were divided into sets using eBURST software (Imperial College, London, UK) (*18*), which is available at the MLST database. eBURST sets meet the requirement that all STs must be single-locus variants (SLVs) of at least 1 other ST within the group. Founder STs were defined as described previously (*18*). Clonal complexes (CCs) consisted of eBURST sets or eBURST sets plus related STs that shared 5 of 7 allelic identities with most other STs included within an eBURST set.

### Antimicrobial Drug Susceptibility Testing and Detection of *erm*B/*mef*A Genes

MICs of serogroup 19 were determined for 6 antimicrobial drugs (penicillin, cefotaxime, chloramphenicol, tetracycline, clindamycin, and erythromycin) by E-test (AB Biodisk, Solna, Sweden) (*19*). Susceptibilities to vancomycin and trimethoprim-sulfamethoxazole were determined by disk diffusion test. Multidrug resistance was defined as nonsusceptibility to >3 antimicrobial drug classes. The *mef*A and *erm*B genes were detected by PCR using the primers *mef*A (forward, 5′-AGT ATC ATT AAT CAC TAG TGC-3′; reverse, 5′-TTC TTC TGG TAC TAA AAG TGG-3′) and *erm*B (forward, 5′-GAA AAG GTA CTC AAC CAA ATA-3′; reverse, 5′-GTA ACG GTA CTT AAA TTG TTT AC-3′) (*20*). PCRs were performed with 35 amplification cycles: 30 s at 94°C, 30 s at 50°C, and 1 min 30 s at 72°C, followed by a final extension at 72°C for 10 min.

### Statistical Analysis

Statistical analysis was performed by using SPSS software version 13.0 (SPSS, Chicago, IL, USA). Serotype proportion in each period was compared using the χ^2^ or Fisher exact test, as appropriate. The Mantel-Haenszel χ^2^ test was used for trend analysis.

## Results

### Changes in Serotype Distributions

From 1991 through 2006, 538 strains of *S. pneumoniae* were obtained from various clinical specimens. Of these, 158 (29%) were from invasive isolates; 124 blood, 15 cerebrospinal fluid, 6 pleural fluid, 5 ascites, and 8 other sterile deep-seated tissues (e.g., bone and joint fluid). The remaining 380 (71%) were from noninvasive isolates; 110 (pharyngeal swab), 91 (transtracheal aspirate), 81 (sputum), 69 (middle ear fluid), 15 (urine), and 14 (open pus).

**Figure 1:**
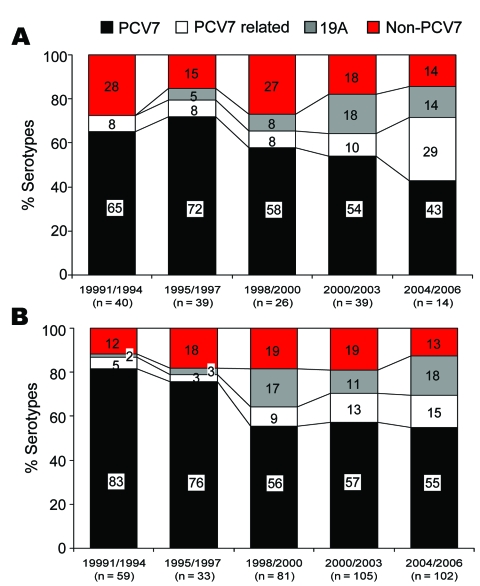
Distribution of serotypes with regard to 7-valent conjugate vaccine (PCV7) among 538 isolates encountered during five 3-year periods from 1991 through 2006, South Korea. A) Invasive isolates. B) Noninvasive isolates.

The most common serotypes were 19F (113, 21%), 23F (96, 17.8%), 19A (58, 10.8%), 6B (50, 9.3%), 6A (43, 8%), 14 (40, 7.4%), and 9V (24, 4.5%); these 7 serotypes accounted for 79% of the total isolates. Overall, PCV7 serotypes accounted for 64.1% of total isolates and 62.7% of invasive isolates. Table 1 shows the serotype distributions of invasive and noninvasive isolates by age group. For invasive isolates, PCV7 serotype coverage was 67% among children <60 months of age and 47% among children >60 months (Table 1). During 2001–2003, just before PCV7 was introduced in South Korea, overall PCV7 coverage rates for PCV7 serotypes and PCV7-related serotypes were 54% and 10% among the 138 invasive isolates and 57% and 13% among the 380 noninvasive isolates. The proportion of serotype 19A isolates increased from 0% (0/40) during 1991–1994 to 18% (7/39) during 2001–2003 among the 138 invasive isolates. Similarly, from 1995–1997 to 1998–2000, the proportion of serotype 19A isolates increased from 3% (1/33) to 17% (14/81) among noninvasive isolates (Figure 1).

**Table 1:** Distributions of serotypes among 538 isolates from children in South Korea, by age group, 1991–2006*

Serotype	No. (%) invasive isolates†					No. (%) noninvasive isolates†			
	<24 mo	24–59 mo	>60 mo	Total		<24 mo	24–59 mo	>60 mo	Total
PCV7 serotypes	44 (67)	28 (68)	24 (47)	96 (61)		113 (65)	81 (71)	42 (45)	236 (62)
19F	8 (12)	6 (15)	4 (8)	18 (11)		50 (29)	33 (29)	12 (13)	95 (25)
23F	16 (24)	6 (15)	4 (8)	26 (16)		32 (18)	20 (18)	18 (19)	70 (18)
6B	8 (12)	3 (7)	7 (14)	18 (11)		19 (11)	10 (9)	3 (3)	32 (8)
14	7 (11)	10 (24)	5 (10)	22 (14)		8 (5)	7 (6)	3 (3)	18 (5)
9V	4 (6)	3 (7)	3 (6)	10 (6)		4 (2)	8 (7)	2 (2)	14 (4)
4	0 (0)	0 (0)	0 (0)	0 (0)		0 (0)	2 (2)	4 (4)	6 (2)
18C	1 (2)	0 (0)	1 (2)	2 (1)		0 (0)	1 (1)	0 (0)	1 (0)
PCV7-related serotypes	3 (5)	6 (15)	7 (14)	16 (10)		15 (9)	14 (12)	9 (9)	38 (10)
6A	3 (5)	5 (12)	6 (12)	14 (9)		12 (7)	10 (9)	7 (7)	29 (8)
23A	0 (0)	1 (2)	1 (2)	2 (1)		3 (2)	4 (4)	0 (0)	7 (2)
9	0 (0)	0 (0)	0 (0)	0 (0)		0 (0)	0 (0)	2 (2)	2 (1)
19A	11 (16)	2 (5)	0 (0)	13 (8)		27 (16)	8 (7)	10 (11)	45 (12)
Non-PCV7 serotypes	8 (12)	5 (12)	20 (39)	33 (21)‡		18 (11)	11 (10)	32 (35)	61 (16)
Total	66 (100)	41 (100)	51 (100)	158 (100)		173 (100)	114 (100)	93 (100)	380 (100)

*PVC7, 7-valent conjugate vaccine. †Percentages have been rounded. ‡Thirteen serogroups were included among 33 invasive isolates as follows: 15 (6 strains), 24F (6), 10 (4), 34 (3), 35 (3), 1 (2), 3 (2), 12F (2), 5 (1), 11A (1), 13 (1), 20 (1), and 27 (1).

**Figure 2:**
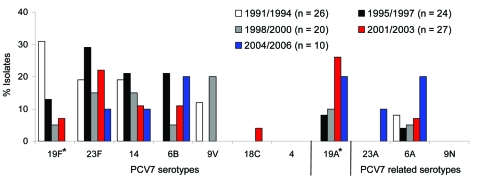
Distribution of serotypes with regard to 7-valent conjugate vaccine (PCV7) among 107 pneumococci isolated from children <5 years of age with an invasive pneumococcal infection (IPD) during five 3- or 4-year periods from 1991 through 2006, South Korea. *The observed increase in the proportion of 19A (p = 0.005) and decrease in the proportion of 19F (p = 0.008) among invasive isolates were statistically significant.

Among the 107 invasive isolates from children <5 years of age (Figure 2), serotype 19F decreased from 31% (8/26) in period 1 to 13% (3/24) in period 2 and to 5% (1/20) in periods 3 and 4 (p = 0.008 for trend). The proportion of 19A increased from 0% (0/26) in period 1 to 8% (2/24) in period 2 and reached 26% (7/27) in period 4 (p = 0.005 for trend). There were no significant trends for the remaining serotypes: 23F (p = 0.58), 14 (p = 0.28), 9V (p = 0.23), 6A (p = 0.38), and 6B (p = 0.23). During period 5, after vaccine introduction, the proportion of 19A isolates was 20% (2/10). During 2001–2003, 19A was the most common serotype among IPD isolates from South Korean children <5 years of age. During 2004–2006, the postvaccine period, when PCV7 uptake reached ≈20%–25% of South Korean children <2 years of age (*21*), the distribution of serotypes was unaltered compared with distribution during the prevaccine 2001–2003 period. There was no major change in antimicrobial drug treatment policy or pressure to use antimicrobial drugs in the local pediatric practice during the study period.

### MLST Analysis of Serogroup 19

MLST analysis showed 4 STs among the 58 serotype 19A isolates: ST320 (52 isolates), ST 1374 (4), ST1451 (1), and ST2394 (1). Eighteen STs were found among the 68 serotype 19F isolates: ST271 (14 isolates), ST320 (6), ST236 (14), ST283 (7), ST1464 (10), ST2395 (3), ST2695 (3), and 1 isolate each of ST1203, ST1412, ST1417, ST2396, ST2397, ST2398, ST2399, ST2694, ST2696, ST2697, and ST2698. Through the course of this study, 11 new STs (ST2394-ST2399 and ST2694-ST2698) were identified among the serogroup 19 isolates, and 8 of these were SLVs or double-locus variants of ST271.

**Figure 3:**
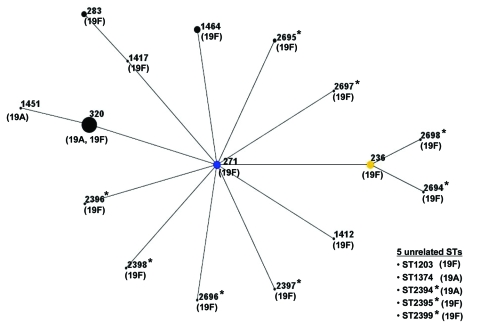
Relationship of 126 strains of serogroup 19 by eBURST analysis. ST271 (blue circle) is the predicted primary founder and ST236 (yellow circle) was assigned to a subgroup founder. Numbers on the diagram correspond to sequence types (STs). The size of each circle correlates with the number of isolates of that ST. *Newly identified ST in this study.

According to eBURST analysis, 1 major CC (CC271) accounted for most of the isolates (n = 116, 92% of serotypes 19A and 19F). CC271 comprised 16 STs, where ST271 was predicted as the founder, and ST320 was the predominant allelic profile. The 5 ST lineages that were unrelated to CC271 were nonlinked singlets (ST1203, ST1374, ST2394, ST2395, and ST2399) (Figure 3).

**Figure 4:**
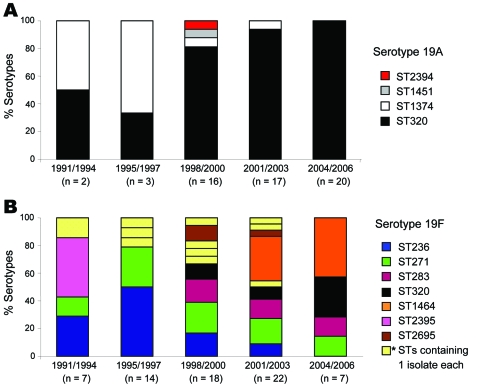
Distributions of sequence types (STs) of serotypes 19A and 19F during five 3-or 4-year periods from 1991 through 2006, South Korea. A) Invasive isolates. B) Noninvasive isolates. *Indicates 11 different STs that contained 1 isolate of serotype 19F.

ST320 was the only ST found among serotypes 19A and 19F; ST320 was the most common ST (n = 52, 90% of total 19A isolates) among serotype 19A. ST320 was observed in only 9% (n = 6) of serotype 19F isolates. The genetic structure of serotype 19A comprised primarily ST1374 during periods 1 and 2 (1991–1997), but ST1374 isolates were not recovered after 2001. In contrast, ST320 was the most common sequence type from 1998, and all 19A isolates from 2002–2006 were of ST320. The numbers of 19A isolates increased consistently from 1996 through 2003, which suggests that single clonal expansion of ST320 was responsible for the increase of serotype 19A isolates during this period (Figure 4).

Unlike serotype 19A, serotype 19F comprises diverse STs grouped within CC271 (16 STs) and 5 different singlets. All STs, except ST1203, were associated with multidrug-resistant *mef/erm-*containing isolates. The distributions of predominant STs among serotype 19F isolates varied during each study period (Figure 4). For example, ST2395 predominated in period 1 (1991–1994), but ST1464 (an SLV of ST271) predominated in periods 4 and 5 (2001–2006).

### Association of ST with Antimicrobial Drug Susceptibility and *mef*/*erm* Prevalence

Antimicrobial drug susceptibilities were tested for 126 of 171 serogroup 19 isolates. Degrees of resistance to penicillin and cefotaxime differed among isolates containing different STs. In addition, a distinct correlation was found between *mef/erm* prevalence and STs (Table 2). All CC271 isolates (n = 116, 16 different STs) were not susceptible to penicillin and cefotaxime and showed multidrug resistance to >3 antimicrobial drug classes. Erythromycin MICs of CC271 isolates were >256 µg/mL and were all positive for *mef* and *erm*. However, 5 singlets that were unrelated to CC271 showed different patterns of antimicrobial drug susceptibility and presence of *mef/erm* determinants (Table 2). Four serotype 19A isolates representing ST1374 appeared to be less resistant to 2 β-lactam antimicrobial drugs than CC271 isolates. ST 1374 strains were also highly resistant to erythromycin (MIC >256 µg/mL) and were positive for *erm*B only. Strains of ST2394, ST2395, and ST2399 showed a lower degree of erythromycin resistance (MIC = 2–8 μg/mL) than CC271 and ST1374 and contained *mef*A only. One serotype, 19F strain of ST1203, did not express *mef* or *erm*. Serotypes 19A and 19F strains with CC271 were shown to be closely related to the internationally established clone, Taiwan^19F^-14, which is multidrug resistant and carries *mef*A/*erm*B macrolide–resistance determinants defined by the pneumococcal molecular epidemiology network (*22*).

**Table 2:** Antimicrobial susceptibility and *mef* and *erm* prevalence of serogroup 19 pneumococcal isolates from children in South Korea, 1991–2006, according to sequence type

CC or ST* (no. strains)	Serotypes	Multidrug resistance†	MIC_50_ (range) in µg/mL for each antimicrobial drug			*mef/erm* determinants
			Penicillin	Cefotaxime	Erythromycin	
CC271-related‡ (116)	19A, 19F	Yes	1.5 (1.0–3.0)	1.0 (0.75–4.0)	>256 (>256)	*mef+, erm+*
ST1203 (1)	19F	No	0.5	0.25	0.5	*mef*
ST1374 (4)	19A	Yes	0.06 (0.04–0.06)	0.12 (0.09–0.12)	>256 (>256)	*mef*
ST2394 (1)	19A	Yes	1.0	0.5	2.0	*mef+, erm*
ST2395 (3)	19F	Yes	4.0 (4.0)	2.0 (1.0–2.0)	2.0 (2.0)	*mef+, erm*
ST2399 (1)	19F	Yes	0.12	0.25	8.0	*mef+, erm*

*CC, clonal complex; ST, sequence type. †Resistant to at least 3 antimicrobial drug classes. ‡CC271-related sequence types: ST320 (59 isolates), ST271 (14), ST236 (14), ST283 (7), ST1451 (1), ST1464 (10), ST2395 (3), ST2695 (3), and 1 isolate of each of ST1412, ST1417, ST2396, ST2397, ST2398, ST2694, ST2696, ST2697, and ST2698.

## Discussion

We found that before PCV7 introduction in South Korea, the proportion of serotype 19A isolates increased from 0% in 1991 to 26% in 2003 but 19F isolates decreased during the same period. Our study also demonstrated that multidrug-resistant ST320 isolates containing *mef/erm* determinants were responsible for the expansion of serotype 19A.

In the United States, serotype 19A is now the most important cause of IPD by replacement serotypes (*4**,**8**,**23**,**24*). Contrary to what we report for South Korea, the increase in the United States was documented after widespread use of PCV7 (*4**–**6*). Increase in non-PCV7 serotypes, i.e., serotype replacement, has been noted in carriage studies and the pre-licensure clinical trials (*11**,**25**,**26*). After widespread use of PCV7 in young children, replacement of serotypes for IPD has been described for several serotypes in previous studies (*4**–**6*). Of these, an increase in the incidence of IPD cases caused by serotype 19A was quite high. Therefore, the increasing prevalence of IPDs caused by serotype 19A among the vaccine target group is of considerable concern. Our findings of an increase in serotype 19A disease before conjugate vaccine introduction calls into question the role vaccine may play in the emergence of serotype 19A disease and suggests that other factors are important.

Of the factors contributing to the increase in serotype 19A, the MLST findings in our study point to a homogeneous pattern of ST320. ST320 of serotype 19A might have originated from ST271 or ST236 strains that have been prevalent among serotype 19F since 1993 (*20*) or could have been introduced from other countries. Thus, single clonal expansion of ST320, related to a multidrug-resistant internationally prevalent clone, Taiwan^19F^-14 (that also carries *mef*A*/erm*B determinants), was most likely responsible for the prevaccine increase observed for serotype 19A. Antimicrobial drug use may provide selective pressure that would give this highly resistant strain an advantage over other strains. A study in the United States demonstrated an increasing prevalence of *mef*A*/erm*B; 17% percent of 221 children had been colonized by *mef*A*/erm*B containing serogroup 19 pneumococci strains after receiving at least 1 dose of PCV7; the major clonal type was related to Taiwan^19F^-14 (*27*). In Alaska, the increase observed in 19A colonization and IPD seems to be related to CC172 clonal expansion (*8*). In contrast, recent genetic analysis of 19A strains isolated after PCV7 use in the United States showed that diverse mechanisms were involved in the expansion of 19A strains, expansion of preexisting predominant CC199, capsular switching of PCV7 types (4, 6, 14, and 9V), and appearance of multiple unrelated multidrug-resistant CCs among serotype 19A strains (CC271, including ST1451 and ST320, CC156, and CC1296) (*4*). Capsular switching of PCV7 serotypes under selective pressure by vaccine use is one of the mechanisms underlying the expansion of serotype 19A (*4**,**11**–**13**,**28*). However, there is no evidence of capsular switching as a contributing factor to the increase in serotype 19A in our study.

We also found that serotype 19F gradually decreased in proportion and diversified to include several newer descendants that differ from the founder by only 1 or 2 of 7 alleles but ST320 was not a major genetic structure among serotype 19F strains (*18*). Thus, it appears that ST320 has a selective advantage in serotype 19A strains, whereas ST320 did not seem to have expansion merit in the close serotype 19F strains. This finding suggests that the properties of particular clonal or capsular types are likely important determining factors of the potential of pneumococci to influence disease type and severity (*29*,*30*). Further studies are necessary to explain why certain sequence types exhibit different selective pressures according to serotype, even for close serotypes such as 19A and 19F.

This study has several limitations. First, pneumococcal isolates were collected at a single center, and thus, may not represent the national situation. However, no surveillance system has been established for IPD in South Korea. Nevertheless, at least 2 studies have demonstrated a recent increase in serotype 19A isolates among children in daycare centers (3% in 2002 and 11% in 2004) (*14**,**31*). In addition, the number of serotype 19A isolates from children and adults at another tertiary South Korean hospital showed an increase over the same period (*32*). Second, the number of 19A isolates was relatively small, and it is possible that uncommon strains possessing minor clones were not detected. Thus, our finding of a clonal expansion of ST320 among serotype 19A strains may be an overstatement. However, MLST analysis showed that all 7 of the 19A strains from colonized children who were identified from a previous study (*14*) were of ST320 (H.J. Lee, unpub. data).

The present study has implications for future pneumococcal immunization programs. In particular, the demonstration of an increase in 19A before the use of PCV7 suggests that PCV7 vaccination may not be entirely responsible for the observed increase of serotype 19A. Had the vaccine been introduced in 2000, as in the United States, the increase of serotype 19A would have been attributed to serotype replacement after PCV7 introduction. Nevertheless, whether minor multidrug-resistant clones of serotype 19A (appearing after the introduction of PCV7 in the United States) possess an advantage to increase with time is a concern (*23*,*29*).

We demonstrated that multidrug-resistant ST320 strains among serotype 19A have selective advantage in terms of expansion over ST1374, which were less resistant to β-lactams in a country where antimicrobial drug therapy is frequently used. Reports on PCV7 efficacy indicate negligible cross-protection for serotype 19A (*11*). In addition, a recent increase in the antimicrobial drug resistance of invasive 19A isolates and the increase in colonization by serogroup 19 strains carrying *mef*/*erm* determinants raise the possibility of potential increases in the prevalence of this clone. Thus, potential for colonization because of widespread antimicrobial drug use and resistance may interact and provide the selective advantage necessary for serotype expansion, which may be the situation in South Korea. Similarly, the influence of population characteristic dynamics, i.e., HIV infection or poverty and overcrowding, as well as PCV7 introduction, may combine with the necessary factor for serotype replacement and play an important role in serotype expansion, which may be the situation in Alaska. It is too early to determine the effect of PCV7 on expansion of serotype 19A in South Korea. PCV7 was introduced in South Korea in November 2003 when PCV7 serotype coverage was 56% among invasive isolates in children <5 years of age. In 2007, PCV7 coverage is ≈30% of the target group (*21*). At this time it is difficult to predict the effect of low vaccination coverage on serotype expansion/replacement. Therefore, surveillance is essential to monitor antimicrobial drug resistance, serotype expansion, and serotype replacement as early indications of an increase in pneumococcal disease by non-PCV7 or PCV7-related serotypes.
